# Effects of long-term hydrogen intervention on the physiological function of rats

**DOI:** 10.1038/s41598-020-75492-w

**Published:** 2020-10-28

**Authors:** Zhi-ming Xun, Qing-hui Zhao, Yan Zhang, Fang-di Ju, Jin He, Ting-ting Yao, Xiao-kang Zhang, Yang Yi, Sheng-nan Ma, Peng-xiang Zhao, Xiao-yan Jin, Ying-xian Li, Xiao-yang Li, Xue-mei Ma, Fei Xie

**Affiliations:** 1 College of Life Science and Bio-Engineering, Beijing University of Technology, No.100, Pingleyuan, Chaoyang District, Beijing, 100124 People’s Republic of China; 2 Beijing Molecular Hydrogen Research Center, Beijing, 100124 People’s Republic of China; 3 Beijing Chao-Yang Hospital Affiliated To Capital Medical University, Beijing, 100043 People’s Republic of China; 4 Affiliated Bayi Brain Hospital, The Seventh Medical Center of PLA General Hospital, Beijing, 100700 People’s Republic of China; 5 State Key Lab of Space Medicine Fundamentals and Application, China Astronaut Research and Training Center, Beijing, 100094 People’s Republic of China

**Keywords:** Biochemistry, Physiology, Medical research

## Abstract

The potential therapeutic effects of molecular hydrogen (H_2_) have now been confirmed in various human and animal-disease models. However, the effects of H_2_ on the physiological function in a normal state have been largely neglected. Hydrogen-rich water (HRW) intake and hydrogen inhalation (HI) are the most common used methods for hydrogen administration, the difference in the effects between HRW intake and HI remains elusive. In the present study, the body weight and 13 serum biochemical parameters were monitored during the six-month hydrogen intervention, all these parameters were significantly altered by oral intake of HRW or HI. Among the 13 parameters, the most striking alterations induced by hydrogen treatment were observed in serum myocardial enzymes spectrum. The results also showed that the changes in these parameters occurred at different time points, and the alterations in most of the parameters were much more significant in HI than HRW. The results of this study provides the basic data for the mechanism research and application of molecular hydrogen in the future.

## Introduction

Molecular hydrogen (H_2_) is a flammable, colorless, odorless, tasteless, non-toxic and nonmetallic gas at room temperature, which has been historically believed to be a biologically inert gas. The first report on the therapeutic effect of H_2_ was published by Dole et al.^1^. They found that hyperbaric treatment of 97.5% hydrogen gas for 2 weeks could markedly induce tumor regression in mice. However, the potential of H_2_ in medical application has not been vastly explored until the biological effects of low concentration hydrogen were found in 2007. Oshawa et al. reported that inhalation of 1–4% hydrogen gas could significantly ameliorate cerebral ischemia–reperfusion injury by selectively reducing hydroxyl radical and peroxynitrite^2^. Since then, the biomedical interest in hydrogen’s potential for preventive and therapeutic applications has grown exponentially. During the past 12 years, the protective effects of H_2_ have been investigated in a variety of pathological conditions such as cardiovascular disorders, hepatic injuries, neuronal disease, diabetes, metabolic syndrome, etc^3,4^. Although the potential therapeutic effects of H_2_ have now been confirmed in over 170 different human and animal-disease models^4^, the effects of hydrogen therapy, especially long term hydrogen intervention, on the physiological function in a normal state remains largely unknown.

Hydrogen therapy can be delivered via several methods, such as inhaling hydrogen gas, drinking hydrogen-rich water (HRW), injecting hydrogen-rich saline (HRS), taking an H_2_ bath, dropping HRS into the eyes, and increasing the production of intestinal H_2_ by bacteria via non-digestible carbohydrates/certain medications^3^. Among them, oral intake of HRW and inhalation of hydrogen gas are the most common routes of hydrogen therapy in clinical trials, however, there is a lack of detailed comparative study on the effects of these two hydrogen delivering methods at present.

The purpose of the present study was to determine the effects of long term of oral intake of HRW and inhalation of hydrogen gas on the physiological function of healthy rats. Body weight and serum biochemical parameters of the rats were monitored during a period of six months. The results of this study could provide basic data for further research on hydrogen medicine.

## Results

### The effects of hydrogen treatment on body weight

**Figure 1:**
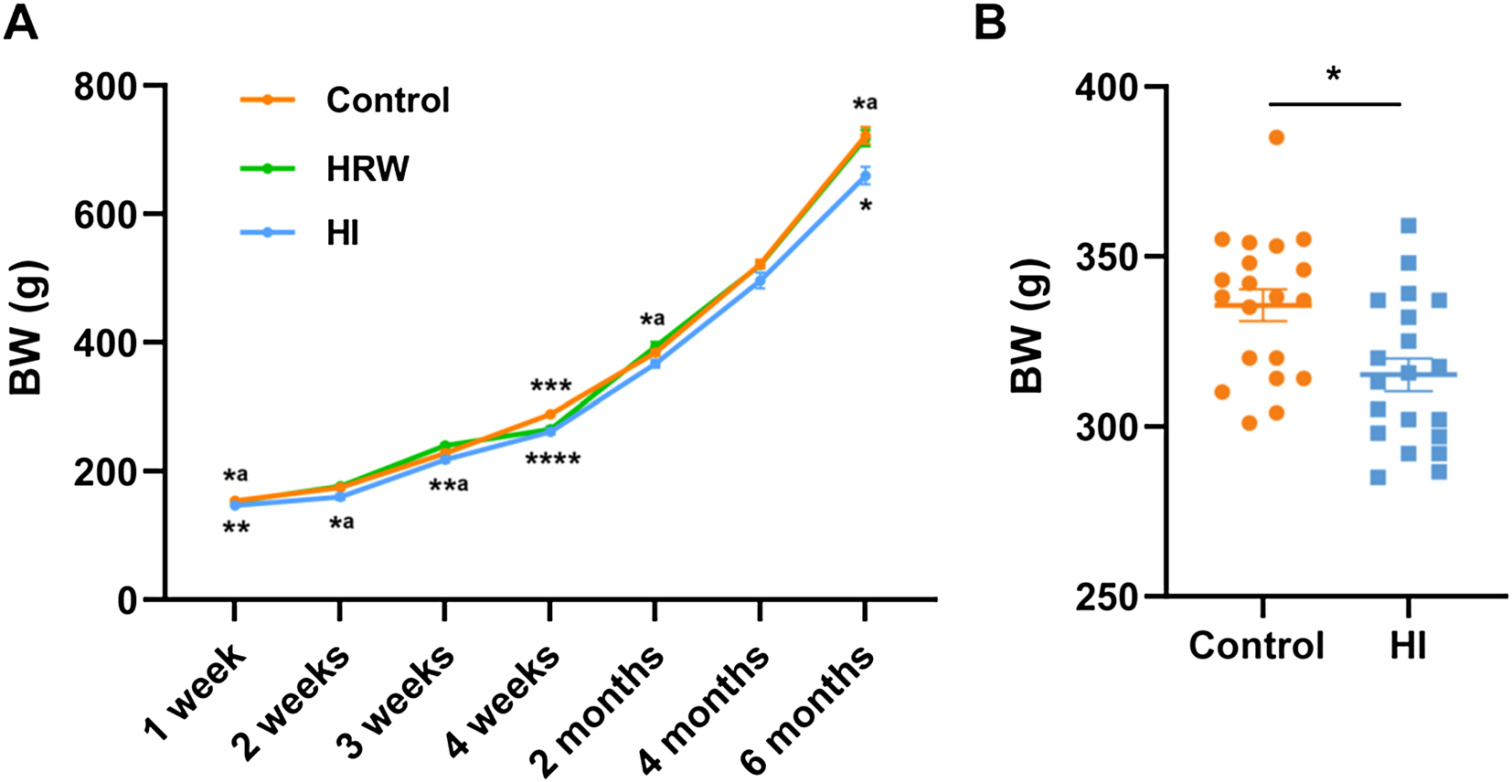
Effects of hydrogen treatment on body weight. (**A**) Body weight of rats in control, HRW and HI group was monitored throughout the six-month period. (**B**) The effect of HI on BW was further confirmed by another experiment in which rats in control and HI group treated with the same operation. Values shown are mean ± SEM (n = 10). For comparison of HRW or HI group and Control group, *p < 0.05, **p < 0.01, ***p < 0.001, ****p < 0.0001. For comparsion of HRW and HI group, *^a^*p* < 0.05, **^a^*p* < 0.01. Figure was made with GraphPad Prism 8.0.2, https://www.graphpad.com/support/faq/prism-802-release-notes.

BW of the rats were monitored during the six-month experimental period. As seen in Fig. 1A, intake of HRW had no significant effect on BW during the six months, except the fourth week (265.64 ± 4.19 g vs. 288.75 ± 2.76 g, *p* = 0.0004). HI decreased the BW from the first week to the end of the study, although the decrease in BW was not significant at some time points (the second week: *p* = 0.070; the third week: *p* > 0.05; the second month: *p* = 0.076; the fourth month: *p* > 0.05). The HI induced BW loss reached a maximum of 8.68% (659.88 ± 13.72 g vs. 722.63 ± 12.76 g, *p* = 0.013) at the sixth month. To exclude the possible effect of placing the rats in the inhalation chamber on BW, we compared additional two groups of rats, including HI group and the Control group treated with the same operation. The results showed that after two weeks of HI, the BW of the rats was markedly lower than the controls (315.10 ± 4.81 g vs. 335.60 ± 4.72 g, *p* = 0.0043) (Fig. 1B).

### The effects of hydrogen treatment on fasting blood glucose

**Figure 2:**
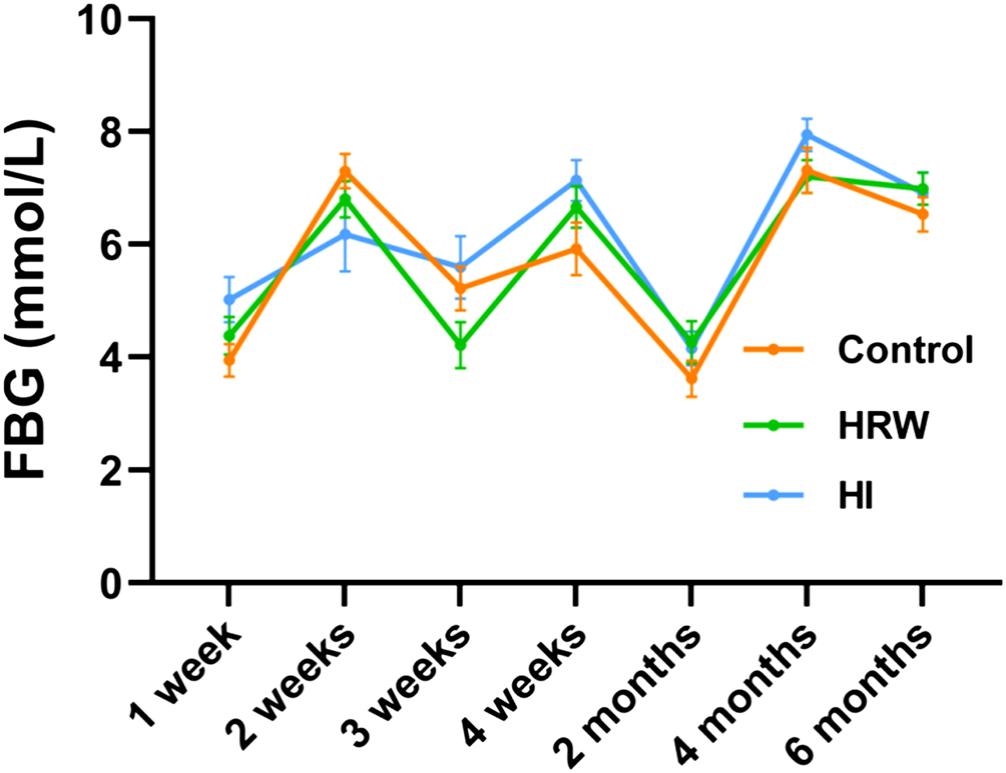
Effects of hydrogen treatment on fasting blood glucose. Values shown are mean ± SEM (n = 10). Figure was made with GraphPad Prism 8.0.2, https://www.graphpad.com/support/faq/prism-802-release-notes.

Fasting blood glucose (FBG) was monitored throughout the study. As seen in Fig. 2, compared with the controls, no statistical difference in FBG levels was observed during the six months either in HRW or HI group.

### The effects of hydrogen treatment on serum uric acid

**Figure 3:**
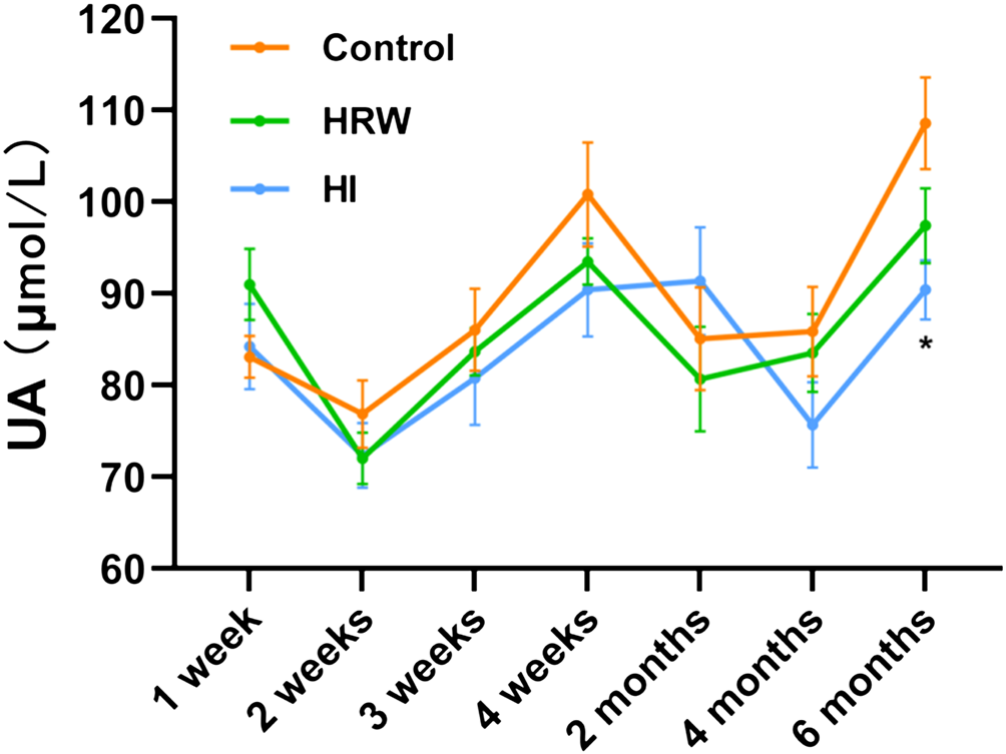
Effects of hydrogen treatment on serum uric acid. Values shown are mean ± SEM (n = 10). For comparison of HI and Control group, *p < 0.05. Figure was made with GraphPad Prism 8.0.2, https://www.graphpad.com/support/faq/prism-802-release-notes.

As seen in Fig. 3, HRW intake had no significant effect on uric acid (UA) levels during the six months. Compared with the controls, there was no statistical difference in UA levels in HI group within the first four months, a significant decrease occurred at the sixth month (90.40 ± 3.21 μmol/L vs. 108.57 ± 5.01 μmol/L, *p* = 0.020).

### The effects of hydrogen treatment on serum lipid and apolipoprotein

**Figure 4:**
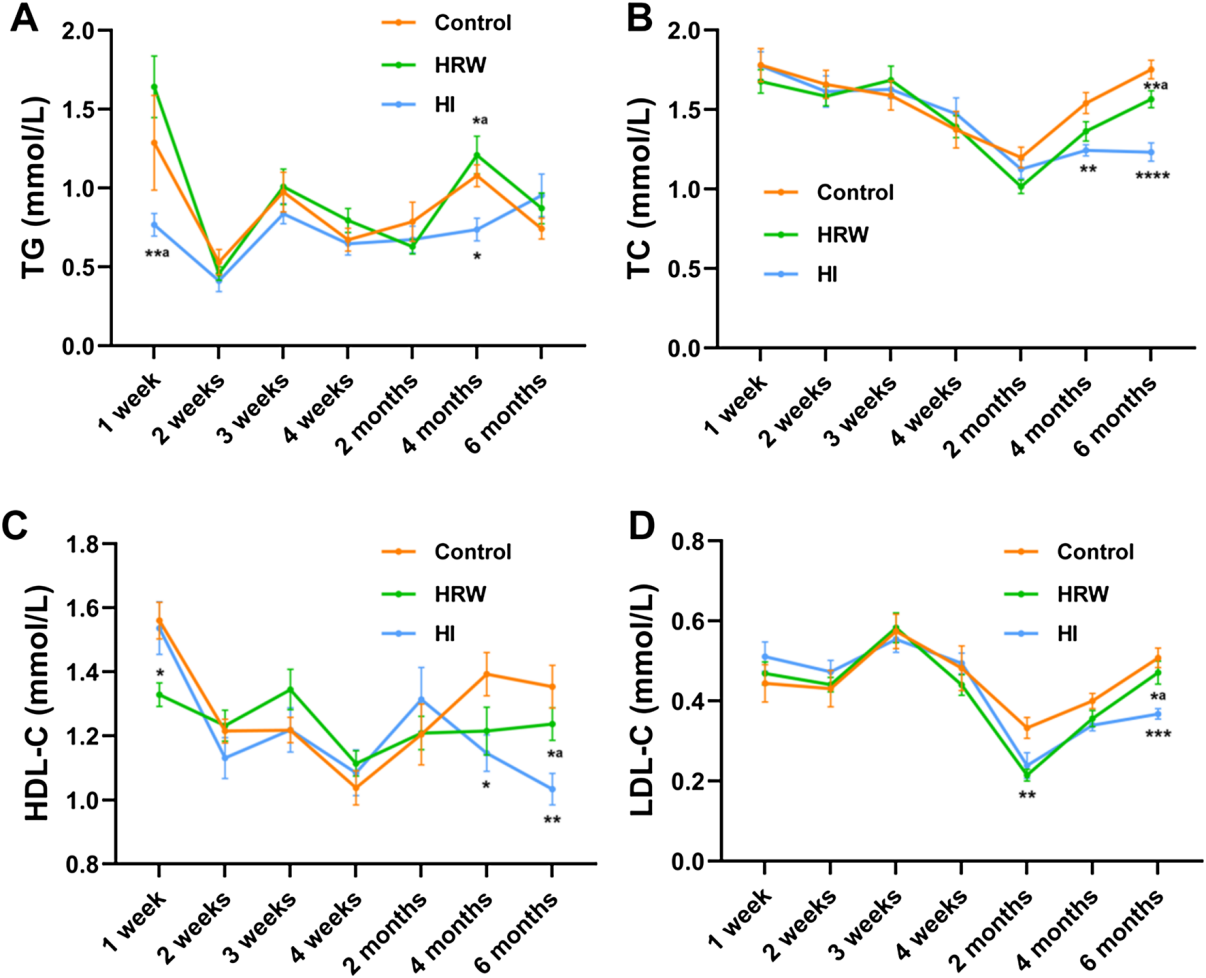
Effects of hydrogen treatment on serum lipid and apolipoprotein. The levels of serum TG **(A)**, TC **(B)**, HDL-C **(C)** and LDL-C **(D)** were monitored throughout the experimental period. Values shown are mean ± SEM (n = 10). For comparison of HRW or HI group and Control group, *p < 0.05, **p < 0.01. Figure was made with GraphPad Prism 8.0.2, https://www.graphpad.com/support/faq/prism-802-release-notes.

There was no significant effect of HRW intake on serum triglycerides (TG) during the six months. Compared the controls, a marked decrease in TG levels occurred at the fourth month (0.74 ± 0.072 mmol/L vs. 1.08 ± 0.069 mmol/L, *p* = 0.012), however, no statistical difference was observed at other time points (Fig. 4A).

HRW intake induced a decline in serum total cholesterol (TC) at the second month (1.02 ± 0.043 mmol/L vs. 1.20 ± 0.065 mmol/L, *p* = 0.075) and the fourth month (1.36 ± 0.061 mmol/L vs. 1.54 ± 0.066 mmol/L, *p* = 0.073) with marginal significance. The decrease was also observed in HI group at the fourth month (1.24 ± 0.035 mmol/L vs. 1.54 ± 0.066 mmol/L, *p* = 0.0077) and the sixth month (1.23 ± 0.057 mmol/L vs. 1.75 ± 0.058 mmol/L, *p* < 0.0001) (Fig. 4B).

The HDL-cholesterol (HDL-C) levels were marked reduced in HRW group at the first week (1.33 ± 0.037 mmol/L vs. 1.56 ± 0.057 mmol/L, *p* = 0.011), however, there was no statistical difference at other time points between HRW group and the controls. HI induced a significant decrease in HDL-C levels at the fourth month (1.15 ± 0.057 mmol/L vs. 1.39 ± 0.067 mmol/L, *p* = 0.039) and the sixth month (1.03 ± 0.049 mmol/L vs. 1.35 ± 0.067 mmol/L, *p* = 0.0036) (Fig. 4C).

HRW intake induced a decrease in LDL-cholesterol (LDL-C) levels at the second month (0.22 ± 0.015 mmol/L vs. 0.33 ± 0.026 mmol/L, *p* = 0.0040), however, there was no statistical difference at other time points between HRW group and the controls. The decline in the LDL-C levels was also observed in HI group from the second to the sixth month (the second month: 0.24 ± 0.032 mmol/L vs. 0.33 ± 0.026 mmol/L, *p* = 0.087; the fourth month: 0.34 ± 0.014 mmol/L vs. 0.40 ± 0.019 mmol/L, *p* = 0.058; the sixth month: 0.37 ± 0.013 mmol/L vs. 0.51 ± 0.024 mmol/L, *p* = 0.0005) (Fig. 4D).

### The effects of hydrogen treatment on liver function indexes

**Figure 5:**
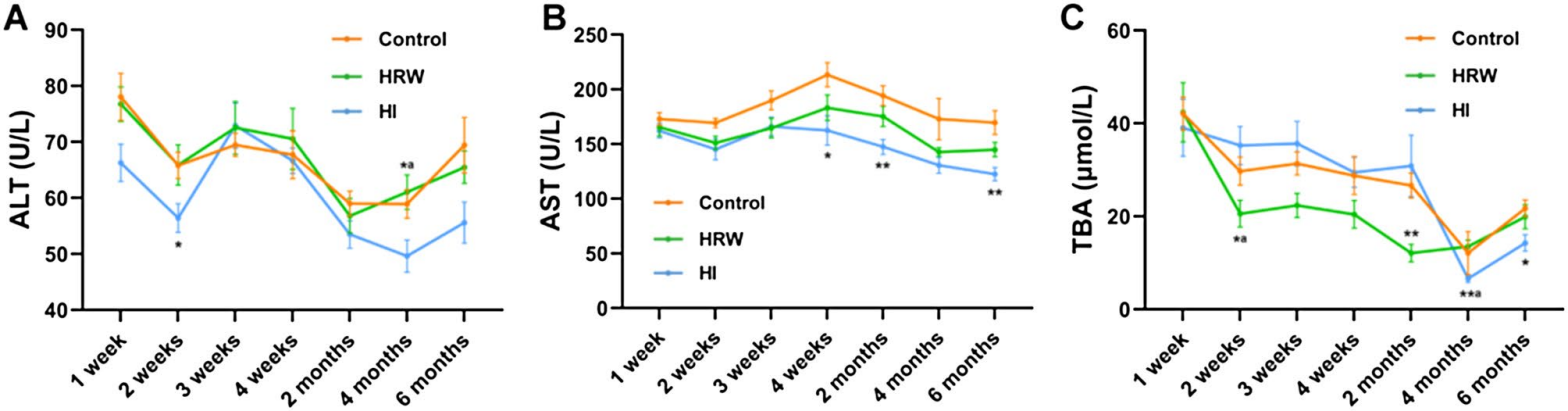
Effects of hydrogen treatment on liver function indexes. The levels of serum ALT **(A)**, AST **(B)** and TBA **(C)** were monitored throughout the experimental period. Values shown are mean ± SEM (n = 10). For comparison of HRW or HI group and Control group, *p < 0.05, **p < 0.01. For comparsion of HRW and HI group, *^a^*p* < 0.05, **^a^*p* < 0.01. Figure was made with GraphPad Prism 8.0.2, https://www.graphpad.com/support/faq/prism-802-release-notes.

There was no significant effect of HRW intake on serum alanine aminotransferase (ALT) levels during the study. Compared with the controls, the serum ALT levels were decreased in HI group at the second week (56.42 ± 2.57 U/L vs. 65.82 ± 2.35 U/L, *p* = 0.039), the fourth month (49.66 ± 2.88 U/L vs. 58.94 ± 2.53 U/L, *p* = 0.074), and the sixth month (55.61 ± 3.67 U/L vs. 69.43 ± 4.93 U/L, *p* = 0.092) (Fig. 5A).

From the second week to the end of the study, the serum aspartate aminotransferase (AST) levels were reduced both in HRW group and HI group. HRW intake decreased AST levels at the second week (145.23 ± 9.51 U/L vs. 169.38 ± 4.27 U/L, *p* = 0.0597) with marginal significance, however, there was no statistical difference at other time points between HRW group and the controls. Compared with the controls, HI induced a significant decrease in AST levels at fourth week (162.52 ± 13.37 U/L vs. 213.47 ± 11.07 U/L, *p* = 0.024), the second month (147.53 ± 6.80 U/L vs. 194.36 ± 9.01 U/L, *p* = 0.0019), and the sixth month (122.64 ± 6.01 U/L vs. 169.70 ± 10.89 U/L, *p* = 0.0053) (Fig. 5B).

From the second week to the second month, the serum total bile acid (TBA) levels in the HRW group were decreased with a maximum decline of 54.58% (12.11 ± 1.88 μmol/L vs. 26.66 ± 2.67 μmol/L, *p* = 0.0011) at the second month. The decline in TBA levels in the HI group occurred at the sixth month (14.29 ± 1.79 μmol/L vs. 21.72 ± 1.81 μmol/L, *p* = 0.026) (Fig. 5C).

### The effects of hydrogen treatment on serum myocardial enzymes spectrum

**Figure 6:**
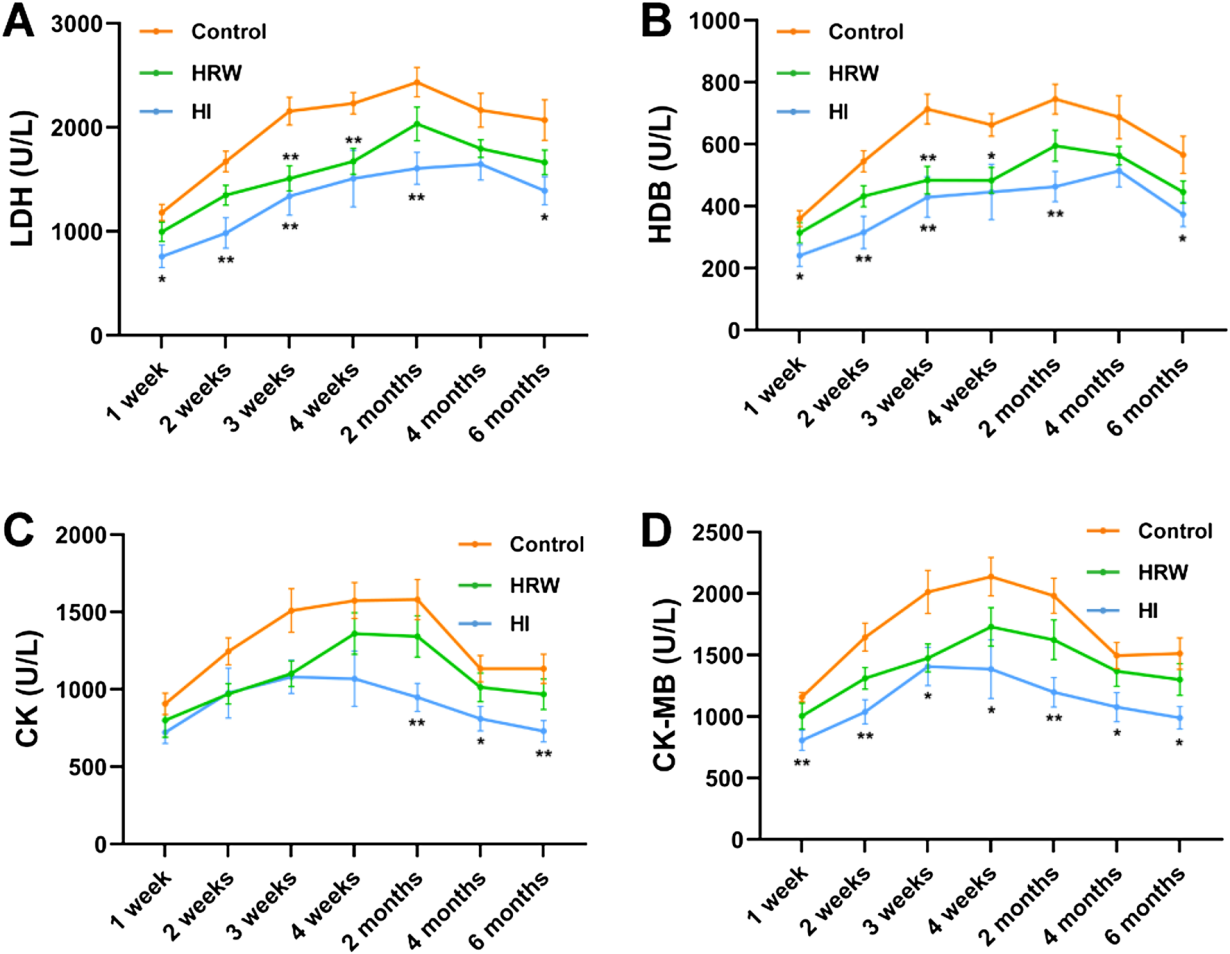
Effects of hydrogen treatment on serum myocardial enzymes spectrum. The activities of serum LDH **(A)**, HDB **(B)**, CK **(C)** and CK-MB **(D)** were monitored throughout the experimental period. Values shown are mean ± SEM (n = 10). For comparison of HRW or HI group and Control group, *p < 0.05, **p < 0.01. Figure was made with GraphPad Prism 8.0.2, https://www.graphpad.com/support/faq/prism-802-release-notes.

The serum activities of myocardial enzymes including lactic dehydrogenase (LDH), α-hydroxybutyric dehydrogenase (HDB), creatine kinase (CK) and its isoenzyme creatine kinase-MB (CK-MB) were monitored throughout the study. The activites of these enzymes were decreased at the first week both in HRW and HI group, and the effect continued to the end of the study. For LDH and HDB activities, the most descending changes occurred at the third week in HRW group (LDH: 1509.83 ± 121.84 U/L vs. 2156.17 ± 131.66 U/L, *p* = 0.0055; HDB: 484.32 ± 44.02 U/L vs. 713.90 ± 48.58 U/L, *p* = 0.0069) and the second week in HI group (LDH: 984.25 ± 146.14 U/L vs. 1672.21 ± 97.90 U/L, *p* = 0.0041; HDB: 315.88 ± 52.26 U/L vs. 545.39 ± 34.36 U/L, *p* = 0.0066) (Fig. 6A,B). For CK and CK-MB activities, the maximum decrease occurred at the third week in HRW group (CK: 1101.70 ± 81.82 U/L vs. 1509.76 ± 140.89 U/L, *p* = 0.061; CK-MB: 1476.83 ± 113.59 U/L vs. 2013.53 ± 175.03 U/L, *p* = 0.052) and the second month in HI group (CK: 948.87 ± 90.69 U/L vs. 1581.31 ± 129.73 U/L, *p* = 0.0028; CK-MB: 1198.41 ± 119.44 U/L vs. 1983.25 ± 142.17 U/L, *p* = 0.0015) (Fig. 6C,D).

## Discussion

Until January 2020, more than 1300 scientific publications regarding the biological effects of molecular hydrogen have been published, accumulating evidence from preclinical or clinical studies indicated the therapeutic potential of H_2_ on the progression of organ damage in various types of disease models. However, most of these studies have focused on the protective roles of H_2_ in pathological conditions, the effects of H_2_ on the physiological function in a normal state have been largely neglected. In the present study, the BW and 13 serum biochemical parameters were monitored during the six-month hydrogen intervention, all these parameters were significantly altered by oral intake of HRW or HI. Firstly, changes in these parameters occurred at different time points. Some parameters, such as TG, HDL-C, ALT, LDH, HDB, CK and CK-MB, changed as early as one week after hydrogen intervention. The impact of hydrogen treatment on UA, TC and LDL-C occurred at relatively late time points. Secondly, the two intervention methods induced a slightly different pattern of change in these parameters. Alterations in most of the parameters, such as BW, UA, TG, ALT, AST, LDH, HDB, CK and CK-MB, were much more significant in HI than HRW, only the changes in TBA were more evident in HRW group. Previous study has shown that HI induces increased hydrogen concentration in rat blood and organ tissues more slowly than intake of HRW, however, HI maintains hydrogen concentration for a longer period^5^, which may partly contribute to the greater impact on most biochemical parameters induced by HI than intake of HRW. Although there are some reference for the normal range of the biochemical parameters in healthy SD rats^6,7^, the normal reference values may be influenced by some factors, such as sex, age, weight, diet, the measuring instruments and methods, etc. The hydrogen induced changes in more than half of the biochemical parameters were less than 30%, these alterations should be within the normal range of healthy rats. TG, TBA, and the myocardial enzymes spectrum changed to a greater extent, however, these alterations may not induce negative effects on health.

Previous studies on the effect of H_2_ in diabetes mellitus (DM) have given inconclusive results. Administration by HRW intake^8^, HRS injection^9^, or subcutaneous injection of hydrogen gas^10^ showed great efficiency in lowing FBG in animal models of DM. However, other studies showed oral intake of HRW could not significantly alter the FBG levels in both animal model^11^ and clinical trial^12^. Our results showed that no significant alterations in FBG both in HRW and HI group during the six months, which was consistent with some of previous studies.

Our results showed a significant decrease in UA, however, this decline occurred as late as six months and only in HI group. To the best of our knowledge, there is only one paper reported the serum UA lowering effect of H_2_^13^, which might be due to the delayed response of serum UA by hydrogen treatment.

The serum lipid and apolipoprotein were significantly altered by hydrogen intervention in our study. The contents of TC and LDL-C were decreased from the second month to the end of the study in both HRW and HI group. The decline in these two parameters after hydrogen treatment were also reported in metabolic syndrome (MS) in other studies^14,15^. Our results also showed that HDL-C levels in HRW group were decreased at the first week, since then returned to the control levels. The levels of HDL-C in HI group were significantly decreased as late as 4 months. The effects of hydrogen treatment on HDL-C were inconsistent in previous studies. Some studies showed that hydrogen intervention had no effect on HDL-C in DM^5,11,12^ and MS^15^. Other studies showed increased levels of HDL-C after hydrogen treatment in DM^10^ and MS^14^. Such inconsistent results in HDL-C might be related to the different detection windows or species in these studies. HRW intake has been shown no effect on TG levels in our study, which was consistent with the previous studies in both DM^12^ and MS^14–16^. HI has been shown significantly decreased the TG levels in our study, this lowering effect on TG was also observed by injection of hydrogen gas^10^ or HRS^11^ in previous reports, which indicated that the effect on TG may be related to the absorption of H_2_ into the body.

Molecular hydrogen has been shown to have a protective role in liver function in liver disease, as evidenced by the decreased ALT, AST or TBA levels^17–19^ in serum. The decline in these parameters were also observed in our study, although ALT was not significantly altered in HRW group. These results indicated that H_2_ may have protective effect in liver function in normal state.

Previous studies showed that H_2_ administration protects against myocardial infarction^20–23^, heart failure^24,25^, toxin-induced myocardial injury^26,27^ and improves cardiac function induced by hypertension^28,29^ and transplantation injury^30,31^. The most striking changes were observed in serum myocardial enzymes spectrum in our study. Both HRW intake and HI induced a decrease in serum LDH, HDB, CK and CK-MB activities at the first week, the alterations continued to the end of the study, and the effects in HI group were much more evident. These evidence indicated that H_2_ may improve cardiac function in normal state. The mechanism underlying the protective effect of H_2_ on cardiac function need to be further investigated.

The limitation of our study may be the lack of monitoring of the food and water consumption, as well as defecation and urination in rats. Considering HI has a significant impact on the BW of rats, the food and water uptake may be influenced by HI. Another limitation of this study is related to lack of measurement of the hydrogen concentration in the blood. Some parameters, such as TG and HDL-C, changed early and the changes gradually disappear, this may be explained by relatively reduced hydrogen uptake with the rapid increase of BW. Some parameters, such as TC and LDL-C, changed as late as two months after hydrogen intervention. Previous study has shown that long-term hydrogen intervention may exert modulatory effects on gut flora components^32^. The slow response to hydrogen may be mediated by the alterations of the gut microbiota, although the detailed mechanism needs to be further investigated.

In conclusion, this study provides the basic data for the mechanism research and application of molecular hydrogen in the future. The effects of different hydrogen administration routes on healthy human subjects needs to be further investigated, which could provide a solid foundation for hydrogen medicine.

## Materials and methods

### Animals and experimental design

Thirty 3-week-old male Sprague–Dawley rats weighing 40–50 g were purchased from Vital River Laboratory Animal Technology Co., Ltd (Beijing, China). Animals were maintained under standard conditions at 22 °C to 25 °C with a 12 h light–dark cycle and were fed a normal diet. All experimental protocols were approved by the Animal Care and Use Committee of Capital Medical University and were conducted in accordance with the Regulations for the Administration of Affairs Concerning Experimental Animals (China). Prior to the experiment the animals were adapted to laboratory conditions for one week. The rats were then randomly divided into three groups (10 in each group): (1) Control group: rats were maintained under normal conditions; (2) HRW group: rats were given HRW by oral intake for 1 h each time, two times per day; (3) HI group: rats were treated with hydrogen inhalation (4%) for 1 h each time, two times per day. The body weight (BW) of the rats was evaluated every week. To exclude the possible effect of placing the rats in the inhalation chamber on BW, forty male SD rats (180–200 g) were randomly divided into two groups: control and HI group, 20 rats in each group. Rats in the control group were treated with the same operation as HI group to inhale the placebo gas (air containing 21% O_2_). BW was determined after two-weeks of hydrogen inhalation.

### Hydrogen rich water preparation

HRW was produced as described in previous study^33^. Briefly, a metallic magnesium stick (Doctor SUISOSUI; Friendear Inc., Tokyo, Japan) was placed into double distilled water for more than 6 h at room temperature. The hydrogen concentration was monitored by using a hydrogen electrode (Unisense A/S, Aarhus, Denmark) every week, and the stick was replaced every two weeks, ensured that the concentration of dissolved hydrogen was maintained above 600 µM.

### Inhalation of hydrogen gas

Animals were placed in a transparent closed box (72 × 53 × 45 cm, length × width × height) connected to the hydrogen gas generator which composed of an Oxy-Hydrogen Machine (SG-3000; Gang’an Health Management [Beijing] Co., Ltd., Beijing, China) and a gas mixer, allowed to spontaneous respiration (4% H_2_, 96% air containing 21% O_2_ ) for 1 h each time, and two times a day. The concentration of hydrogen and oxygen in the closed box was monitored by Thermal trace GC ultra-gas chromatography (Thermo Fisher, MA, USA).

### Biochemical assay

Blood samples (about 300 µl per rat) were harvested by eyeball blood collection in the morning after an overnight fast. Serum was obtained by centrifuging for 20 min at 1000 g. FBG, UA, TG, TC, HDL-C, LDL-C, ALT, AST, TBA, LDH, HDB, CK and its isoenzyme CK-MB were measured by commercial kits (Nanjing Jiancheng Biochemistry, China) according to the manufacturer’s instructions. The measurements were performed by using a Victor 1420 Multilabel Counter (Wallac, Perkin-Elmer, Wellesley, MA, USA).

### Statistical analysis

All statistical analyses were performed using GraphPad Prism 8.0.2. To exclude the possible effect of placing the rats in the inhalation chamber on BW, comparison analysis on BW between Control and HI groups were determined by Student's *t* test. All other data were analyzed by two-way ANOVA repeated measures with Tukey’s post hoc test. Data were shown as the mean ± SEM. A value of *p* < 0.05 was considered significant.

## Data Availability

The data that support the findings of this study are available from the corresponding author upon reasonable request.
